# Using Multigroup-Multiphase Latent State-Trait Models to Study Treatment-Induced Changes in Intra-Individual State Variability: An Application to Smokers' Affect

**DOI:** 10.3389/fpsyg.2016.01043

**Published:** 2016-07-22

**Authors:** Christian Geiser, Daniel Griffin, Saul Shiffman

**Affiliations:** ^1^Department of Psychology, Utah State UniversityLogan, UT, USA; ^2^Department of Psychology, University of PittsburghPittsburgh, PA, USA

**Keywords:** smokers' affect state variability, latent state-trait models, multigroup confirmatory factor analysis, nicotine replacement therapy, ecological momentary assessment (EMA)

## Abstract

Sometimes, researchers are interested in whether an intervention, experimental manipulation, or other treatment causes changes in intra-individual state variability. The authors show how multigroup-multiphase latent state-trait (MG-MP-LST) models can be used to examine treatment effects with regard to both mean differences and differences in state variability. The approach is illustrated based on a randomized controlled trial in which *N* = 338 smokers were randomly assigned to nicotine replacement therapy (NRT) vs. placebo prior to quitting smoking. We found that post quitting, smokers in both the NRT and placebo group had significantly reduced intra-individual affect state variability with respect to the affect items *calm* and *content* relative to the pre-quitting phase. This reduction in state variability did not differ between the NRT and placebo groups, indicating that quitting smoking may lead to a stabilization of individuals' affect states regardless of whether or not individuals receive NRT.

In intervention, evaluation, and experimental studies, researchers are interested in testing the effects of an intervention, program, or experimental manipulation on one or more outcome variables (for simplicity and in order to save space, we hereafter refer to interventions, programs, experimental manipulations, etc., simply as “treatment”). Frequently, such studies include multiple groups (e.g., one or more treatment and control groups) that are compared in order to determine the magnitude and statistical significance of a potential treatment effect. Classical designs are control/treatment pretest-posttest designs in which individuals are either randomly assigned to treatment and control groups (true experimental design) or non-randomly assigned (e.g., when individuals self-select into groups or are members of pre-existing groups; quasi-experimental design). The pretest allows researchers to check for pre-existing differences between groups (due to e.g., “unhappy” randomization or a quasi-experimental design), whereas the comparison of pretest and posttest differences between groups allows testing the significance and size of a potential treatment effect.

In many experimental and quasi-experimental studies, researchers focus on mean differences between (a) groups and (b) across time [i.e., mean changes from the pre- to posttest(s)]. Using smoking cessation treatment as an example from which we draw our illustrative dataset, researchers have demonstrated that mean negative affect levels generally increase when smokers first quit, and that treatment with nicotine replacement therapy (NRT) results in smaller changes in negative affect levels (Shiffman et al., 2006). Standard statistical methods for testing mean differences include uni- and multi-variate analysis of (co)variance ([M]AN[C]OVA) and multigroup confirmatory factor analysis (MG-CFA, Jöreskog, 1971). An advantage of MG-CFA over (M)AN(C)OVA techniques is that the MG-CFA analysis uses latent variables so that researchers can explicitly account for and quantify the degree of measurement error and test for *latent* mean differences (Bollen, 1989). Furthermore, the MG-CFA approach is more flexible in terms of its assumptions (e.g., with regard to whether homogeneity of variances across groups needs to be assumed) and in terms of testing assumptions. For example, the assumption of measurement equivalence (ME) across groups pre and post treatment is implicitly made (but not formally tested) within the (M)AN(C)OVA framework, whereas this assumption can be tested within the MG-CFA framework via model fit statistics.

In the present paper, we present extensions of current multivariate latent variable techniques for group comparisons in pre-post experimental (or quasi-experimental) designs when researchers are interested in whether a treatment caused changes not (or not only) in the latent means, but in the degree of *state variability*. With *state variability*, we mean systematic intra-individual fluctuations of people's true scores across time. More formally, within the framework of latent state-trait (LST) theory described in detail below, state variability refers to deviations of so-called latent state scores from so-called latent trait scores. For example, it is well-known that people's mood levels fluctuate to a certain extent across time (e.g., Eid et al., 1999). Some people may suffer not only from low average positive mood levels (e.g., due to a dysthymic personality), but also from large mood level fluctuations (e.g., due to a bipolar disorder). As a consequence, a treatment may be developed to (1) increase patients' average mood levels and (2) reduce the degree of intra-individual mood fluctuations so that individuals' mood levels become more stable intra-individually after treatment.

Changes in state variability are frequently of interest to researchers in addition to studying mean changes (Hedeker et al., 2009). As another example, consider the following research hypothesis pursued in the substantive-empirical portion of the present article. Based on a randomized controlled trial (RCT), Shiffman et al. (2006) were able to show that NRT can have a significant positive effect on smoker's mean affect levels after quitting smoking as compared to a placebo group. In Shiffman et al.'s (2006) study, smokers who received a nicotine patch in the quitting phase on average reported significantly higher positive affect and significantly lower negative affect compared to a placebo group within the first 3 days of abstinence.

What has not been tested so far is whether NRT can also have a positive effect in terms of decreasing intra-individual affect *variability*, that is, on fluctuations in state affect around individuals' stable trait affect levels. In other words, in addition to improving individuals' overall trait (mean) affect, NRT might be hypothesized to also have a “stabilizing effect” in terms of reducing the extent to which individuals' affect states fluctuate across time after quitting. This might be expected because nicotine withdrawal is thought to make (ex-)smokers more emotionally volatile or irritable, that is, more reactive to affective provocation (Acri and Grunberg, 1992).

Consider the following hypothetical example (illustrated in Figure 1): Assume that an individual, say John, reports the same general (i.e., average) true affect level of seven points on a 10-point Likert scale pre and post quitting. Although John has the same general affect level of seven pre and post quitting, he shows greater variability of his affect states around this general level post quitting as compared to the pre-quitting phase. In the example in Figure 1, John's true scores fluctuate between 5 and 8 pre-quitting, whereas his true scores fluctuate more dramatically between 2 and 10 post quitting. One could say that John's affect level has become more “state-like” (less stable) post-quitting. In the present study, we demonstrate how extended latent state-trait (LST) models can be used to test hypotheses with regard to treatment-induced changes in state variability. To our knowledge, our paper is the first to demonstrate the use of LST models for this purpose.

**Figure 1:**
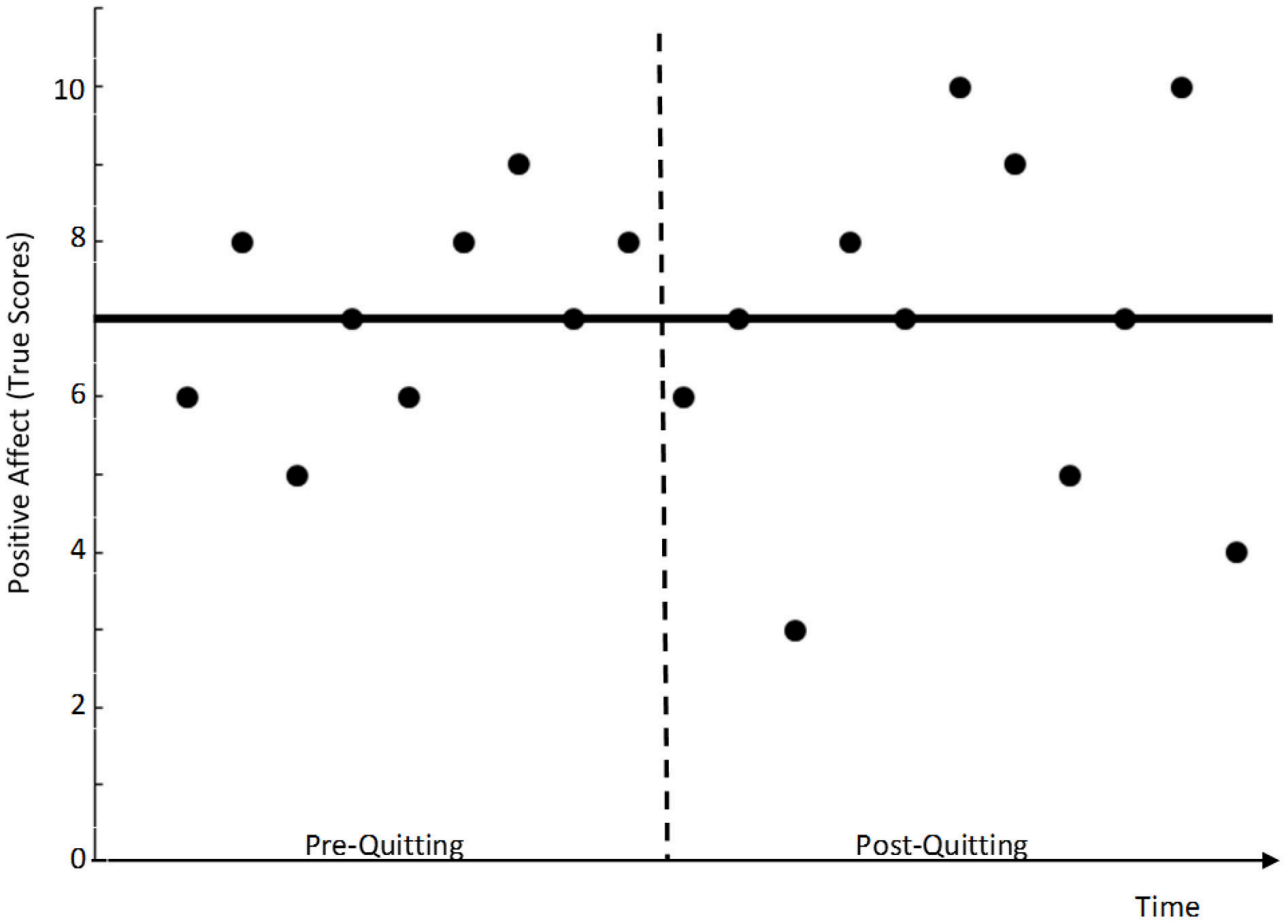
**Hypothetical example of a smoker who shows increased intra-individual state variability of his affect true scores after quitting smoking**. The solid bar indicates the intra-individual true score mean, which did not change from the pre- to the post-quitting phase.

Substantively, we tested the hypotheses that (1) smokers who quit smoking would generally show increased intra-individual state variability with regard to their affect post quitting as compared to a pre-quitting phase and that (2) smokers in an NRT group would show significantly less strongly increased intra-individual mood variability levels after quitting compared to smokers in a placebo group.

The purpose of the present paper is twofold. From a methodological perspective, we show how multigroup-multiphase latent state-trait (MG-MP-LST) models can be used to test treatment effects in terms of differential changes between groups in (1) latent trait means and (2) the amount of intra-individual state variability. The MG-MP-LST models presented here are extensions of multigroup LST models presented by Steyer et al. (2015). From a substantive perspective, we test whether NRT has a positive effect not only on smokers' mean positive mood (as was shown by Shiffman et al., 2006), but also on smokers' mood state variability (i.e., whether NRT leads to a reduction in mood variability) after they quit smoking.

## LST theory and models

LST theory and models have been developed to take systematic intra-individual variability of human behavior into account in the measurement of psychological attributes and to separate stable (i.e., trait) from variable (situation-specific) components of behavior and random measurement error (Steyer et al., 1992)^1^. According to LST theory, intra-individual variability in measurements across time can be due to (1) long-term changes in the trait values, (2) short-term situation-specific fluctuations (state variability), or (3) random measurement error (Bishop et al., 2015). Models of LST theory allow researchers to disentangle these three sources of variability and to quantify the proportion of variability in an observed variable that is due to (1) stable trait factors and/or trait changes, (2) fluctuating state residual variability, and (3) random measurement error.

**Figure 2:**
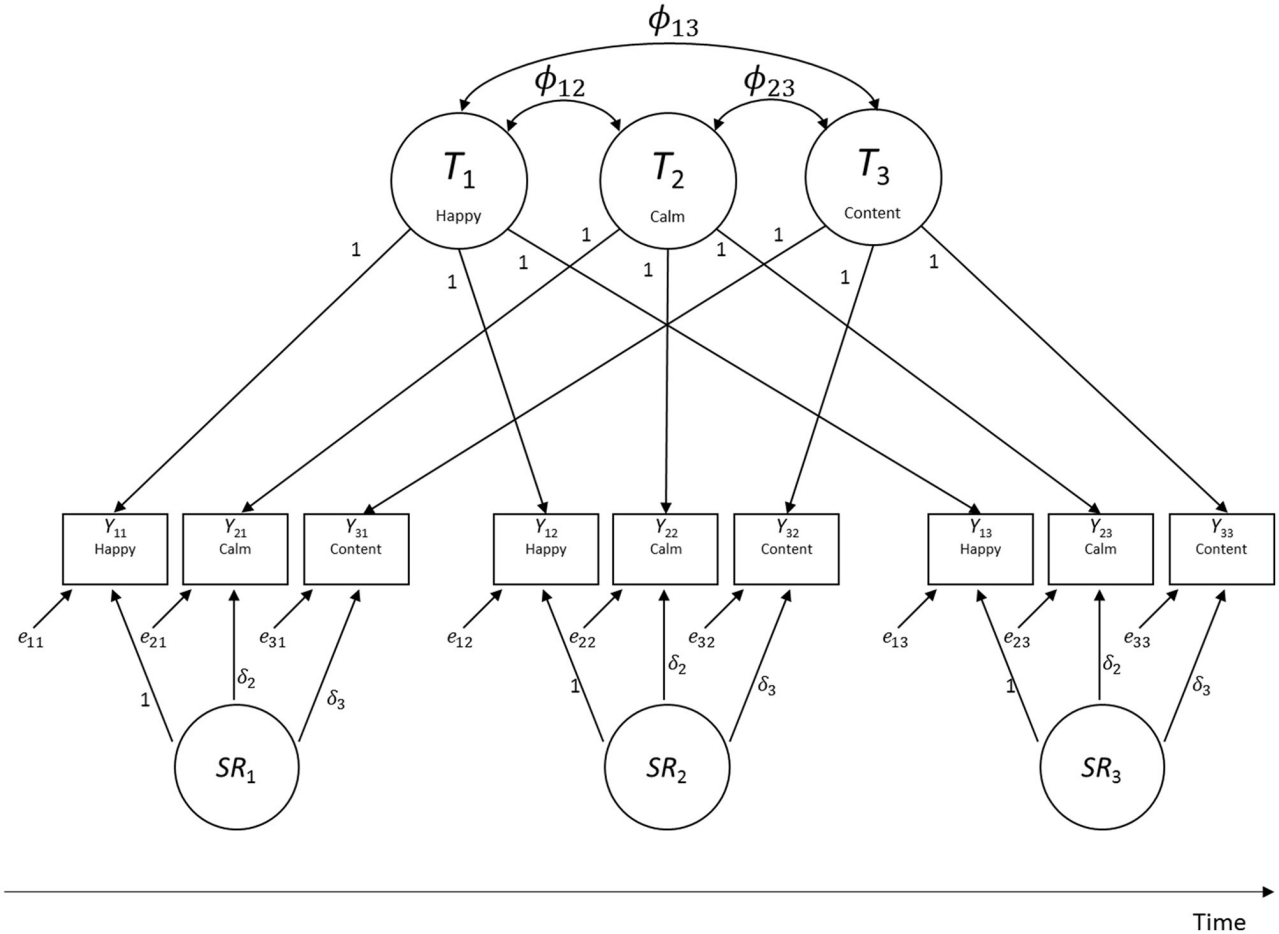
**Path diagram of a multitrait-multistate (MTMS) model of LST theory for three indicators measured on three time points**. The parameters ϕ_*ij*_ indicate covariances between indicator-specific trait factors.

To illustrate the basic structure of LST models, Figure 2 shows a path diagram of an LST model with indicator-specific trait factors (Eid, 1996). In this example, three observed variables (indicators *Y*_*it*_, *i* = indicator, *t* = time point) were measured on three time points. In the figure, *T*_*i*_ indicates a latent trait factor pertaining to indicator *i*. *T*_*i*_ reflects the stable component of behavior or emotion that does not vary across occasions of measurement with regard to indicator *i* (e.g., habitual or “trait” happiness). *SR*_*t*_ indicates a latent state residual factor with a mean of zero that characterizes systematic residual variability at time *t* (i.e., individuals' true situation-specific deviations from their stable trait values; e.g., deviations of state mood from trait mood). The variances (*Var*) of the *SR*_*t*_ factors thus capture the amount of state variability in a given construct. When *Var*(*SR*_*t*_) = 0 for all *t*, there is no state variability, indicating that a construct is perfectly trait-like. The variables *e*_*it*_ indicate unsystematic measurement error in the sense of classical test theory (CTT; Novick, 1966) and thus have zero means by definition as well.

The model in Figure 2 is referred to as multitrait-multistate (MTMS) model, because it uses as many trait factors *T*_*i*_ as there are indicators. The rational for using as many trait factors as there are indicators is that indicators in the social sciences frequently contain systematic item- or indicator-specific variance that is stable across time. In addition, indicators may measure slightly different traits or distinct facets of the same trait. Because of this heterogeneity, models with just a single trait factor typically do not fit empirical data well (Geiser and Lockhart, 2012). For example, the items *happy* and *calm* may both be viewed as indicators of positive affect, yet they reflect (partly) distinct dimensions or aspects of affect (e.g., *calm* implies a low-arousal state, whereas *happy* does not). The measurement equation for the MTMS model is given by:

(1) $$\begin{array}{l} Y_{it}=T_{i}+\delta_{i}SR_{t}+e_{it}, \end{array}$$

where latent means are estimated for all *T*_*i*_ factors (but no intercepts α_*i*_). All factor loadings on the *T*_*i*_ factors are set to one (i.e., λ_*i*_ = 1 for all *i* and *t*) and there is no constant intercept parameter in the equation. (The rationale for this specification is discussed in detail in Geiser et al., 2015).

In the model, all *T*_*i*_'s are uncorrelated with all *SR*_*t*_ and *e*_*it*_ variables, all *SR*_*t*_ variables are uncorrelated with all *e*_*it*_, and all *e*_*it*_ variables are uncorrelated with each other. Correlations between the indicator-specific trait factors *T*_*i*_ can be estimated. These correlations indicate to which degree indicators are homogenous (measure the same or closely related traits). High correlations indicate homogeneity, moderate to low correlations indicate heterogeneity of indicators. When *Corr*(*T*_*i*_, *T*_*j*_) = 1 for all *i, j*, indicators are perfectly homogeneous so that a simpler LST model with a single trait factor (so-called singletrait-multistate or STMS model) can be used.

In the MTMS and other LST models, temporally adjacent latent state residual variables *SR*_*t*_ are frequently assumed to be connected through a first-order autoregressive structure such that

(2) $$\begin{array}{l} SR_{t}=\beta_{t}SR_{t-1}+\zeta_{t}, \end{array}$$

where β_*t*_ indicates a regression slope coefficient and ζ_*t*_ indicates a latent residual variable with a mean of zero that is uncorrelated with all other latent and error variables in the model (Cole et al., 2005).

Modeling an autoregressive structure as in Equation (2) can be necessary when measurement occasions are closely adjacent in time; in this case, there might be carry-over effects such that the deviation from the trait score at time *t* depends in part on the deviation at the previous time point *t*−1. For example, mood deviations from a general mood level are typically stable for at least a few hours. Such dependencies are less likely, however, when time intervals between measurement occasions are larger. In practice, researchers can empirically test whether autoregressive effects are present by estimating both the MTMS model with and without an autoregressive process and compare the fit of the two models. If there is no difference in model fit between the two model versions as indicated by a chi-square difference test for nested models, then the researcher would prefer the simpler model without autoregressive path coefficients.

### Variance components and coefficients

The MTMS model allows for an additive decomposition of the variance of the observed and latent true score variables (see formulas in Table 1). According to the MTMS model, observed variance *Var*(*Y*_*it*_) is the (weighted) sum of trait, state residual, and error variance. True score variance *Var*(τ_*it*_) is given as the (weighted) sum of trait and state residual variance (i.e., excluding random error variance). Note that the larger the trait variance relative to total true score variance, the more “trait-like” a given construct; in contrast, the larger the state residual variance relative to total true score variance, the more “state-like” a given construct (and the larger the amount of state variability).

**Table 1:** **Variance decomposition and coefficients in the MTMS Model**.

**Variance/coefficient**	**Equation**
Observed variance	*Var*(*Y*_it_**) = *Var*(τ**_it_**) *+ Var*(*e*_it_**)
True score variance	*Var*(τ*_it_*) = *Var*(*T_i_*) + δ*_i_*^2^*Var*(*SR_t_*)
Consistency *CO*	*CO*(τ*_it_*) = *Var*(*T_i_*)/*Var*(τ*_it_*)
Occasion-specificity *OS*	*OS*(τ*_it_*) = δ*_i_*^2^*Var*(*SR_t_*)/*Var*(τ*_it_*)
Reliability *Rel*	*Rel*(*Y_it_*) = *Var*(τ*_it_*)/*Var*(*Y_it_*) = [*Var*(*T_i_*) + δ*_i_*^2^*Var*(*SR_t_*)]/*Var*(*Y_it_*)

*Y_it_, observed variable (i, indicator, t, time point). τ_it_, true score variable. e_it_, measurement error variable. T_i_, indicator-specific trait factor. SR_t_, state residual factor. λ_i_, trait factor loading. δ_i_, state residual factor loading*.

Based on the additive variance decomposition, coefficients can be defined that formally represent these different proportions of variability. The consistency coefficient *CO* gives the proportion of variance that is accounted for by trait factors. *CO* thus measures the degree of stability (“situation-independence”) of a psychological construct. The occasion-specificity coefficient *OS* gives the proportion of variance that is accounted for by state residual factors. *OS* thus measures the degree of situation-dependence (state variability) of a psychological construct. The reliability coefficient *Rel* gives the proportion of observed variance that is not due to random measurement error. *Rel* thus has the same meaning as in CTT. The trait and state residual variance components as well as the *CO* and *OS* coefficients are of particular relevance to the present study. Below we explain how an extended version of the MTMS model for multigroup pre-post designs can be used to study treatment-related changes and group differences in these variance components.

### Extended LST models for multigroup-multiphase designs

Traditionally, LST models such as the MTMS model shown in Figure 2 have mostly been applied to single-group longitudinal data without a treatment (e.g., pretest-posttest) component. For example, Wu (2016) applied LST models to depression self-report measures to determine the extent to which such measures reflected a stable trait vs. fluctuating state construct. In the present paper, we show how extended MG-MP-LST models can be used to study changes in (1) trait values and (2) intra-individual state variability based on a multigroup-multiphase design when researchers have collected data on multiple time points both *pre* and *post* treatment in two or more groups.

Steyer et al. (2015) presented MG-MP-LST models that can be used in experimental and quasi-experimental pre-post designs with multiple groups. Whereas Steyer et al. (2015) focused on the use of MG-MP-LST models to examine treatment-induced changes in trait means, in the present paper we demonstrate the use of these models for studying changes in state and trait variability.

**Figure 3:**
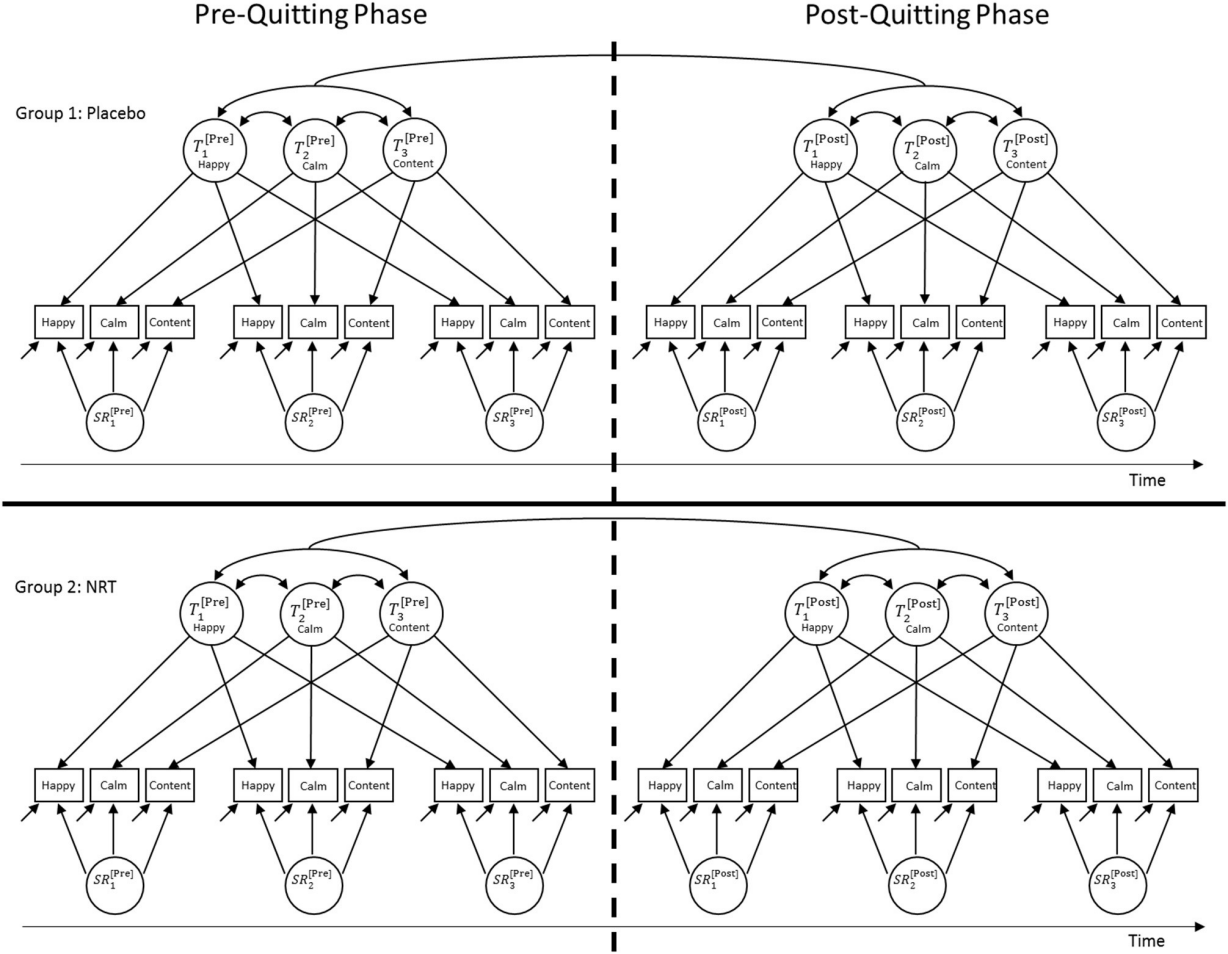
**Path diagram of a multigroup-multiphase-(MG-MP-)LST model for three indicators measured on three time points pre- and post an intervention (here: quitting smoking) in two groups (here: placebo vs. NRT)**. All indicator-specific trait factors can be correlated within and across phases.

A MG-MP-LST model with indicator-specific traits (MTMS approach) is illustrated in Figure 3. There are two key differences between the conventional MTMS model described above and the MG-MP-LST model. First, the parameters of MG-MP-LST models are estimated simultaneously in two or more groups in line with multigroup CFA (Jöreskog, 1971). The multigroup approach allows for group comparisons with regard to (a) measurement-related parameters such as factor loadings, intercepts, and measurement error variances to test for ME across groups (e.g., Widaman and Reise, 1997; Millsap, 2011) and (b) structural parameters such as latent factor means, variances, and covariances. Second, as discussed in more detail below, MG-MP-LST models estimate separate (but correlated) trait factors for different phases, for example, (1) a pre- vs. (2) a post-treatment phase.

Figure 3 illustrates an MG-MP-LST model for a design with three indicators, three measurement occasions in the pre- and post-treatment phase, respectively, and two groups (i.e., a placebo = control group and an NRT = treatment group). Note that extensions of the model to more indicators, time points, or groups are straightforward. It can be seen that in the MG-MP-LST model there are separate indicator-specific trait factors for (1) the pre- and (2) the post-treatment phase. Therefore, individuals' trait scores in the pre-treatment phase need not be the same as the trait scores in the post-treatment phase. This reflects the assumption that a treatment might cause (more enduring) changes to individuals' latent trait scores, potentially resulting in changes in the trait means or variances.

For example, quitting smoking without NRT might cause a decrease in the trait factor mean for the item *happy* post-quitting in the placebo group, indicating an overall decrease in positive trait mood in this group, due to nicotine withdrawal, or removal of positive-affect-enhancing effects of nicotine use. In addition, quitting without NRT may cause a decrease in the trait factor variance for the same item (relative to the total true score variance), indicating an overall decrease in the intra-individual stability of this trait (i.e., the construct may have become less trait-like as a result of quitting as mood becomes more volatile).

Furthermore, the model contains separate latent state residual factors for each measurement occasion within the pre- and post-treatment phase. Note that similar to the trait factor variances, the variances of the state residual factors can vary across the pre- and post-treatment phases. A change in the amount of latent state residual factor variance from pretest to posttest (relative to total true score variance) would indicate changes in the amount of intra-individual state variability. For example, the amount of state residual factor variance for mood items may increase relative to the total true score variance from the pre- to post-quitting phase. This would indicate that a construct has become less stable (i.e., less trait-like and more state-like) as a result of quitting and/or NRT. Given that the latent state residual factors are defined as regression residual factors in LST theory (Steyer et al., 1992, 2015), these factors have zero means by definition in each group. Mean changes from the pre- to the post-quitting phase can (and need) only be assessed for the trait factors in the model.

The model in Figure 3 uses indicator-specific trait factors. Therefore, this model allows for the possibility that each item could show differential changes with regard to the trait means or with regard to the amount of trait and state variability across the pre- and post-quitting phases. For example, the responses to the item *happy* may be more or less affected by quitting and/or NRT than responses to the items *calm* or *content*. This is an advantage of the model compared to models with a single general trait factor, which implicitly assume that all indicators are similarly affected by potential treatment effects.

## Parameter invariance testing across groups and time

A general advantage of the multigroup CFA modeling approach over (M)AN(C)OVA methods is the flexibility with which the multigroup CFA approach allows testing various measurement and structural (latent variable) parameters of interest for invariance across groups. In this way, numerous hypotheses with regard to pre/post and group differences can be tested via model (goodness-of-fit) comparisons. The most parsimonious model (i.e., the model that shows the highest level of parameter invariance across groups —i.e., “group equality”) that still shows an appropriate fit is typically selected. The model comparisons allow researchers to make statements about the extent to which (1) the groups differ with regard to measurement and/or structural parameters before and after the treatment, (2) whether there are changes in measurement and/or structural parameters from the pre- to post-treatment phase within each group, and (3) whether treatment groups showed stronger changes in relevant parameters from the pre- to post-treatment phase compared to control groups.

For meaningful comparisons of latent variable variances across groups and time, factor loadings should be equivalent across groups and time (Widaman and Reise, 1997). In the case of the MG-MP-LST model, this means that for a meaningful comparison of state residual factor variances across groups and time, the state residual factor loadings should be invariant across groups *g* and phases (i.e., $\delta_{ig}^{\text{PRE}}=\delta_{ig^{\prime }}^{\text{PRE}}=\delta_{ig}^{\text{POST}}=\delta_{ig^{\prime }}^{\text{POST}}$ for all *i, g*). For meaningful comparisons of latent variable means, both factor loadings and intercept parameters should be equivalent across groups and time. Note that in the MTMS model all intercept parameters are implicitly zero and all trait factor loadings are equal to 1 by definition (Geiser et al., 2015). Therefore, invariance of the intercepts and trait factor loadings across groups and time is implied in the MTMS model. In addition to the state residual factor loadings, researchers can test whether measurement error variances are equivalent across time and groups.

## Materials and methods

### Sample

We re-analyzed data from *N* = 338 smokers who participated in a randomized controlled trial, in which smokers were randomly assigned to a placebo group (*n* = 147) or an NRT group (*n* = 191). In the quitting phase, participants in the NRT group received nicotine patches, whereas placebo group members received placebo patches. More details on the sample, recruitment process, and procedure are described in Shiffman et al. (2006).

### Measures and procedure

Smokers provided self-reports of their affect via ecological momentary assessments (EMA) for a period of about 2 weeks prior to quitting (pre-quitting phase) as well as for a period of about 6 weeks after quitting (post-quitting phase). For the present methodological illustration, we selected three items measuring positive affect (*happy, content, calm*; Cronbach alpha = 0.8) that were assessed on a scale from 0 (minimal positive affect) −10 (maximal positive affect). Given the large number of response categories, we treated the items as continuous variables in the analyses.

For the present application and illustration of MG-MP-LST models and to simplify the analysis, we selected three measurement occasions in the pre-quitting phase and three measurement occasions in the post-quitting phase from each participant's responses to the three affect items. The pre-quitting data recording period lasted for 16 days. On Day 9, participants practiced quitting (“trial abstinence period”; Shiffman et al., 2006). We therefore selected data from the first 8 days within the pre-quitting phase. This allowed us to avoid the potential effects that trial abstinence might have on smoker's affect as well as a potential affect disturbance during the days directly before the actual quitting phase due to an anticipation of withdrawal symptoms. In order to allow participants to acclimate to the EMA devices we excluded all data recorded on the first day of measurement in the pre-quitting phase. The approximate time interval between the measurement occasions included in the present analysis was ~2–3 days for both the pre- and post-quitting phase.

We also excluded data from the first day of abstinence (the first day of the post-quitting phase) in order to avoid bias due to the initial affect disturbance immediately after quitting, and to allow time for the effects of NRT to become evident. All measurements taken from reports of smoking during the pre- or post-quitting phase were excluded from the analysis. That is, we used only randomly prompted non-smoking occasions pre-quitting and excluded data from participants after a relapse post quitting (for participants who experienced a relapse at some point within the post-quitting phase, we still included all pre-quitting data as well as all post-quitting data *prior* to the relapse in the analysis). The Mplus script for specifying the final model can be found in the online Supplementary Materials.

## Results

In order to examine potential effects of NRT on smoker's mood variability, we used MG-MP-LST analysis (Steyer et al., 1992, 2015) as described above. In the first step of our analysis, we estimated separate models within each group and phase. This was done for several reasons. First, this allowed us to test whether a model with indicator-specific traits (MTMS approach) was needed or whether a simpler model with a general trait factor (STMS approach) could be used. Second, it allowed us to check whether the inclusion of an autoregressive structure (Equation 2) was necessary. Third, estimating separate models within each group and phase allowed us to test for ME within and across phases within each group. In summary, these preliminary analyses allowed us to make informed decision as to which model should be used for the combined multigroup-multiphase analysis.

Our preliminary analyses revealed statistically significant indicator-specificity (i.e., the trait components of the items *happy, calm*, and *content* did not follow a unidimensional structure). This was shown by the fact that models with a single trait factor (STMS approach) fit significantly worse than models with indicator-specific traits (MTMS approach) in both groups and phases. We therefore used the MTMS approach in all subsequent analyses. Furthermore, our preliminary analyses revealed that the inclusion of autoregressive effects was not necessary as indicated by non-significant chi-square difference tests for comparisons of model versions with and without autoregressive effects as well as non-significant β_*t*_ coefficients. We therefore used an MTMS approach without autoregressive structure in our final combined MG-MP-LST model. Finally, the preliminary analyses revealed that invariant latent state residual factor loadings (δ) could be assumed within phases within each group, so that we used models with time-invariant δ-parameters within phases in the combined MG-MP-LST analysis. Error variances were found to be non-invariant across time, so that we left the error variances unconstrained in subsequent analyses.

Table 2 shows model goodness-of-fit tests and descriptive indices for all versions of the combined MG-MP-LST model that we tested. RMSEA values < 0.05 as well as CFI values > 0.95 are commonly seen as indicative of an appropriate model fit (Hu and Bentler, 1999). Model comparisons were based on chi-square difference tests (with non-significant chi-square difference values indicating that a more restricted model did not fit worse than the more general model that it was compared to) and AIC values (with smaller AIC values indicating better fit). Model 1 represented the least restrictive version of the model in Figure 3. In Model 1, all free parameters (i.e., parameters that are not fixed by definition of the model) were allowed to vary between the placebo and NRT groups. Specifically, Model 1 allowed the state residual factor loadings, measurement error variances, trait factor variances, trait factor means, and trait factor covariances as well as the state residual factor variances to differ between groups. Model 1 thus served as an unrestrictive baseline model. This model showed a reasonably good fit to the data as indicated by the RMSEA and CFI so that we used this model for subsequent invariance tests across groups.

**Table 2:** **Model goodness-of-fit measures for different MG-MP-LST models with indicator-specific trait factors fit to smoker's affect data**.

**Model**	**χ^2^**	***df***	***p***	**RMSEA**	**CFI**	**AIC**	**χ^2Δ^**	***df*Δ**	***p*(χ^2^Δ)**
1. δ*_i_* loadings free across groups	381.37	268	< 0.001	0.05	0.95	20049			
2. δ*_i_* loadings equal across groups	394.49	274	< 0.001	0.05	0.94	20051	13.12	6	0.004
3. δ*_i_* loadings equal across groups except for *content*	383.61	273	< 0.001	0.05	0.95	20042	2.24	5	0.82
4. Equal *SR_t_* variances *within* each group and phase	395.46	281	< 0.001	0.05	0.94	20037	11.85	8	0.16
5. Equal *SR_t_* variances across groups *within each phase*	400.77	283	< 0.001	0.05	0.94	20039	5.31	2	0.07
6. All *SR_t_* variances equal	406.25	284	< 0.001	0.05	0.94	20042	5.48	1	0.02
7. Equal *T_i_* variances across groups within each phase	405.84	289	< 0.001	0.05	0.94	20032	5.07	6	0.53
8. Equal *T_i_* variances across groups and phases	419.03	292	< 0.001	0.05	0.94	20039	13.20	3	0.004
9. Equal *T_i_* variances across phases only for *happy*	408.01	290	< 0.001	0.05	0.94	20032	2.18	1	0.14
10. Equal *T_i_* variances across phases for *happy* and *calm*	415.85	291	< 0.001	0.05	0.94	20038	7.84	1	0.01
11. Equal *T_i_* variances across phases for *happy* and *content*	414.13	291	< 0.001	0.05	0.94	20036	6.11	1	0.01
12. Equal *T_i_* means across groups within each phase	423.60	296	< 0.001	0.05	0.94	20036	15.59	6	0.02
13. Equal *T_i_* means across groups only pre-quitting	413.53	293	< 0.001	0.05	0.94	20032	5.52	3	0.14
14. Equal *T_i_* means across phases only in placebo group	417.43	296	< 0.001	0.05	0.94	20029	3.90	3	0.27
15. Equal *T_i_* covariances across groups within each phase	427.93	302	< 0.001	0.05	0.94	**20028**	10.49	6	0.11

*RMSEA, root mean square error of approximation; CFI, comparative fit index; AIC, Akaike's information criterion. Bold face indicates lowest AIC value*.

In Model 2, we constrained all latent state residual factor loadings (δ) to be equal across groups. The chi-square difference test for the comparison between Model 2 and Model 1 was significant, indicating that the assumption of equal factor loadings for all items was too restrictive. A closer investigation of the individual loadings revealed that the unstandardized loadings for the item *content* had dropped significantly in the placebo group from the pre- to the post-quitting phase [from 1.36 (*SE* = 0.14) to 0.39 (*SE* = 0.17)] This may indicate that the situation-specific effects were to a lesser extent shared between this item and the two other items in the placebo group post-quitting. We discuss potential substantive explanations for this phenomenon in the Discussion section.

A model version with partial invariance of the loadings (loadings for *content* left free to vary post quitting in the placebo group only; Model 3) did not fit significantly worse than Model 1 according to the chi-square difference test. Given that the ME assumption was not rejected for the loadings of the two remaining items, we used Model 3 with partial ME in subsequent analyses (partial state residual factor loading invariance is sufficient for meaningful comparisons of state residual factor variances across groups)^2^. With Model 4, we examined whether the state residual factor variances could be assumed to be equal *within* each group and phase, respectively. This model did not impose any across-group or across-phase invariance constraints on the state residual factor variances. Model 4 was useful to test, because if the state residual factor variances could be set equal within each group and phase, this would facilitate the comparison of state residual factor variances across groups and phases and save additional parameters, making the overall model more parsimonious. The chi-square difference test for the comparison between Model 4 and Model 3 was non-significant, indicating that the assumption of equal state residual factor variances within each group and phase was not rejected.

In the next step (Model 5), we tested whether the state residual factor variances were equal across groups *within* each phase (without constraining these variances to be equal *across* the pre- and post-quitting phases). Model 5 did not fit significantly worse than Model 4 indicating that the state residual factor variances could be assumed to be equal across groups within the pre- and post-quitting phases, respectively. Using Model 6, we tested whether *all* state residual factor variances were equal (i.e., whether these variances were not only equal between groups, but also between phases). Model 6 fit significantly worse than Model 5, indicating that the assumption of equal state residual factor variances across the pre- and post-quitting phase was rejected.

Inspection of the estimated state residual factor variances in Model 5 revealed that, contrary to our substantive hypothesis, these variances were *larger* in the pre-quitting as compared to the post-quitting phase, indicating a *decrease* in state variability from pre- to post-quitting in both groups. We therefore used Model 5 for further invariance tests. Model 7 tested whether the trait factor variances were equal across groups within each phase. This assumption was not rejected by the chi-square difference test, as Model 7 did not fit significantly worse than Model 5. In the next step (Model 8), we tested whether the trait factor variances were equal across phases. The chi-square difference test for the comparison between Model 8 and Model 7 was significant, indicating a significant difference in at least some of the trait variances across phases. A closer investigation revealed that the trait variances differed across phases for the items *calm* and *content*, whereas they could be assumed to be equal for the item *happy* (see Table 2, Models 9 through 11).

Model 12 tested whether trait factor means were equal across groups within each phase. This assumption was rejected by the chi-square difference test. In contrast, Model 13, in which the trait factor means were assumed to be equal across groups only in the pre-quitting phase (but not post quitting) did not fit significantly worse than Model 9. In Model 14, we tested whether the trait factor means were equal across phases in the placebo group only. Model 14 did not fit significantly worse than Model 13, indicating that the trait factor means did not change significantly from the pre- to post quitting phase in the placebo group, whereas the post-quitting trait means were significantly larger in the NRT group as compared to the pre-quitting means in this group.

Model 15 tested whether the trait factor covariances could be assumed to be equal across the placebo and NRT groups within the pre- and post-quitting phase. This assumption was not rejected as indicated by a comparison of the chi-square values between Model 15 and 14. We therefore selected Model 15 as our final model.

In summary, the invariance analyses revealed that in both the placebo and NRT groups, the state residual factor variances decreased significantly from the pre- to the post-quitting phase, indicating a significant reduction in intra-individual affect state variability after quitting smoking in both groups. At the same time, the trait factor variances for the items *calm* and *content* increased significantly in both groups from the pre- to post quitting phase, showing that individuals' responses became more stable after quitting. Although, there was a significant mean difference in trait positive affect between groups post quitting (with higher means in the NRT group), the state residual and trait factor variances did not differ between groups (neither pre- nor post quitting). This indicated that NRT did not differentially influence affect variability as compared to placebo treatment.

Table 3 shows the estimated parameters for Model 15. The *CO, OS*, and *Rel* coefficients that were calculated based on the same model can be found in Table 4. Given that the state residual factor variances were set equal within each phase, the items showed the same *CO* and *OS* values within each phase and are thus given only once. Further note that since the trait and state residual factor variances were set equal across groups within the pre-quitting phase, all *CO* and *OS* values are identical across groups pre-quitting (as one would expect in a randomized controlled trial before the treatment phase). Post-quitting, the *CO* and *OS* values are also identical, except for the item *content*, which showed non-invariant loadings from the pre- to post-quitting phase in the placebo group. Because of the drop in the factor loadings, this item has a much higher *CO* value post quitting as compared to the pre-quitting *CO* value. We discuss this issue more in the Discussion Section.

**Table 3:** **Parameter estimates for Model 15**.

**Parameter**	**Placebo group**			**NRT group**		
	**Estimate**	***SE***	**Standardized estimate [range]**	**Estimate**	***SE***	**Standardized estimate [range]**
**PRE-QUITTING PHASE**						
State residual loading *happy* δ^PRE^_1_	1^a^	−^a^	[0.46; 0.48]	1^a^	−^a^	[0.45; 0.46]
State residual loading *calm* δ^PRE^_2_	1.13^b^^,^^c^^,^^e^	0.11^b^^,^^c^^,^^e^	[0.51; 0.52]	1.13^b^^,^^c^^,^^e^	0.11^b^^,^^c^^,^^e^	[0.47; 0.48]
State residual loading *content* δ^PRE^_3_	1.28^b^^,^^e^	0.12^b^^,^^e^	[0.54; 0.59]	1.28*^b^^,^^c^^,^^e^*	0.12^b^^,^^c^^,^^e^	[0.50; 0.58]
Trait loading *happy* λ^PRE^_1_	1^a^	−^a^	[0.66; 0.68]	1^a^	−^a^	[0.64; 0.66]
Trait loading *calm* λ^PRE^_2_	1^a^	−^a^	[0.58; 0.59]	1^a^	−^a^	[0.54; 0.55]
Trait loading *content* λ^PRE^_3_	1^a^	−^a^	[0.57; 0.63]	1^a^	−^a^	[0.53; 0.62]
Error variance *happy Var*(ε^PRE^_1*t*_) [range]	[1.31; 1.69]	[0.28; 0.30]		[1.63; 2.00]	[0.27; 0.32]	
Error variance *calm Var*(ε^PRE^_2*t*_) [range]	[1.81; 2.05]	[0.34; 0.37]		[2.61; 2.79]	[0.30; 0.41]	
Error variance *content Var*(ε^PRE^_3*t*_) [range]	[1.17; 2.19]	[0.30; 0.41]		[1.30; 3.02]	[0.31; 0.47]	
State residual variances *Var*(*SR*^PRE^)	1.00^b^^,^^e^	0.14^b^^,^^e^		1.00^b^^,^^e^	0.14^b^^,^^e^	
Trait variance *happy Var*(*T*^PRE^_1_)	2.02^c^^,^^e^	0.19^c^^,^^e^		2.02^c^^,^^e^	0.19^c^^,^^e^	
Trait variance *calm Var*(*T*^PRE^_2_)	1.68^e^	0.24^e^		1.68^e^	0.24^e^	
Trait variance *content Var*(*T*^PRE^_3_)	1.83^e^	0.25^e^		1.83^e^	0.25^e^	
Trait mean *happy E*(*T*^PRE^_1_)	7.52^c^^,^^e^	0.09^c^^,^^e^		7.52^c^^,^^e^	0.09^c^^,^^e^	
Trait mean *calm E*(*T*^PRE^_2_)	7.39^c^^,^^e^	0.09^c^^,^^e^		7.39^c^^,^^e^	0.09^c^^,^^e^	
Trait mean *content E*(*T*^PRE^_3_)	7.01^c^^,^^e^	0.09^c^^,^^e^		7.01^c^^,^^e^	0.09^c^^,^^e^	
Trait covariance *happy, calm Cov*(*T*^PRE^_1_, *T*^PRE^_2_)	1.21^e^	0.18^e^	0.66^e^	1.21^e^	0.18^e^	0.66^e^
Trait covariance *happy, content Cov*(*T*^PRE^_1_, *T*^PRE^_3_)	1.41^e^	0.18^e^	0.73^e^	1.41^e^	0.18^e^	0.73^e^
Trait covariance *calm, content Cov*(*T*^PRE^_2_, *T*^PRE^_3_)	1.38^e^	0.20^e^	0.79^e^	1.38^e^	0.20^e^	0.79^e^
**POST-QUITTING PHASE**						
State residual loading *happy* δ^POST^_1_	1^a^	−^a^	[0.36; 0.43]	1^a^	−^a^	[0.41; 0.42]
State residual loading *calm* δ^POST^_2_	1.13^c^^,^^d^^,^^e^	0.11^c^^,^^d^^,^^e^	[0.40; 0.47]	1.13^c^^,^^d^^,^^e^	0.11^c^^,^^d^^,^^e^	[0.40; 0.41]
State residual loading *content* δ^POST^_3_	0.66^d^	0.20^d^	[0.24; 0.26]	1.28^c^^,^^d^	0.12 ^c^^,^^d^	[0.46; 0.49]
Trait loading *happy* λ^POST^_1_	1^a^	−^a^	[0.61; 0.73]	1^a^	−^a^	[0.70; 0.70]
Trait loading *calm* λ^POST^_2_	1^a^	−^a^	[0.69; 0.80]	1^a^	−^a^	[0.68; 0.71]
Trait loading *content* λ^POST^_3_	1^a^	−^a^	[0.71; 0.76]	1^a^	−^a^	[0.69; 0.75]
Error variance *happy Var*(ε^POST^_1*t*_) [range]	[1.12; 2.69]	[0.35; 0.57]		[1.37; 1.46]	[0.25; 0.28]	
Error variance *calm Var*(ε^POST^_2*t*_) [range]	[0.62; 1.97]	[0.35; 0.51]		[2.03; 2.18]	[0.37; 0.39]	
Error variance *content Var*(ε^POST^_3*t*_) [range]	[1.65; 2.39]	[0.39; 0.56]		[0.94; 1.72]	[0.26; 0.35]	
State residual variances *Var*(*SR*^POST^)	0.71^d^^,^^e^	0.12^d^^,^^e^		0.71^d^^,^^e^	0.12^d^^,^^e^	
Trait variance *happy Var*(*T*^POST^_1_)	2.02^c^^,^^e^	0.19 ^c^^,^^e^		2.02 ^c^^,^^e^	0.19 ^c^^,^^e^	
Trait variance *calm Var*(*T*^POST^_2_)	2.66^e^	0.32^e^		2.66^e^	0.32^e^	
Trait variance *content Var*(*T*^POST^_3_)	2.66^e^	0.31^e^		2.66^e^	0.31^e^	
Trait mean *happy E*(*T*^POST^_1_)	7.52^c^	0.09^c^		7.81	0.13	
Trait mean *calm E*(*T*^POST^_2_)	7.39^c^	0.09^c^		7.73	0.15	
Trait mean *content E*(*T*^POST^_3_)	7.01^c^	0.09^c^		7.33	0.15	
Trait covariance *happy, calm Cov*(*T*^POST^_1_, *T*^POST^_2_)	1.69^e^	0.21^e^	0.73^e^	1.69 ^e^	0.21^e^	0.7^e^
Trait covariance *happy, content Cov*(*T*^POST^_1_, *T*^POST^_3_)	1.92^e^	0.20^e^	0.83^e^	1.92^e^	0.20^e^	0.83^e^
Trait covariance *calm, content Cov*(*T*^POST^_2_, *T*^POST^_3_)	2.03^e^	0.26	0.76^e^	2.03^e^	0.26	0.76^e^
**ACROSS PHASES**						
Trait covariance *happy* pre-post *Cov*(*T*^PRE^_1_, *T*^POST^_1_)	1.47	0.24	0.73	1.38	0.22	0.68
Trait covariance *calm* pre-post *Cov*(*T*^PRE^_2_, *T*^POST^_2_)	0.90	0.30	0.43	1.38	0.25	0.65
Trait covariance *content* pre-post *Cov*(*T*^PRE^_3_, *T*^POST^_3_)	1.46	0.31	0.66	1.33	0.24	0.60

a *fixed parameter*.

b *Parameter set equal across time within the pre-quitting phase*.

c *Parameter set equal across the pre- and post-quitting phases*.

d *Parameter set equal across time within the post-quitting phase*.

e *Parameter set equal across placebo and NRT groups*.

**Table 4:** **Estimates of consistency, occasion-specificity, and reliability derived from Model 15**.

**Item**	**Placebo group**			**NRT group**		
	***CO*(τ*_i_*)**	***OS*(τ*_i_*)**	***Rel*(*Y_i_*) [range]**	***CO*(τ*_i_*)**	***OS*(τ*_i_*)**	***Rel*(*Y_i_*) [range]**
**PRE-QUITTING PHASE**						
Happy	0.67	0.33	[0.64; 0.70]	0.67	0.33	[0.60; 0.65]
Calm	0.57	0.43	[0.59; 0.62]	0.57	0.43	[0.51; 0.53]
Content	0.53	0.47	[0.61; 0.75]	0.53	0.47	[0.53; 0.73]
**POST-QUITTING PHASE**						
Happy	0.74	0.26	[0.50; 0.71]	0.74	0.26	[0.65; 0.67]
Calm	0.75	0.25	[0.64; 0.85]	0.75	0.25	[0.62; 0.67]
Content	0.90	0.10	[0.55; 0.64]	0.70	0.30	[0.69; 0.80]

*CO(τ_i_), consistency coefficient; OS(τ_i_), occasion-specificity coefficient; Rel(Y_i_), reliability coefficient. For each item, CO(τ_i_) and OS(τ_i_) add up to 1 (100% true score variance). The CO(τ_i_) and OS(τ_i_) coefficients are equal across groups due to the tested invariance constraints, except for the item content due to the non-invariant loadings of this item post quitting*.

As can be seen from the *CO* and *OS* coefficients in Table 4, the item *happy* showed the highest level of stability (trait-like aspects) of the three items in the pre-quitting phase, with about 67% of this item's true score variability being attributable to trait influences and only about 33% of the true score variability being due to situation-specific fluctuations (deviations of individuals' true states from their true trait levels). Post quitting, the item *happy* showed slightly increased stability, with now 74% of its true score variance representing trait variance. Note, however, that the corresponding trait variance component was not statistically significantly larger post-quitting for this item in either group. This result is therefore purely descriptive.

In contrast, both the items *calm* and *content* showed statistically significantly larger trait variance components post quitting in both groups. For *calm*, the percentage of trait variability increased from 57% in the pre-quitting phase to 75% in both groups. The item content showed an increase from 53% trait variance pre-quitting to 90% trait variance in the placebo group and 70% in the NRT group.

## Discussion

In the present article, we illustrated how MG-MP-LST models can be used to study changes in the amount of intra-individual state variability in studies that include a pre- and post-intervention phase. To our knowledge, the present paper is the first to discuss the use of such models to study potential experimental effects on state and trait variability. We presented an empirical application of these models to smokers' affect levels before and after an NRT intervention (vs. placebo). By using models that are derived from LST theory, researchers are able to clearly distinguish between changes in trait mean levels and changes in state variability. Furthermore, researchers can separate out variability that is due to random measurement error. The multigroup modeling approach has the advantage that a number of assumptions (such as ME across time and groups) can be formally tested through nested model comparisons. This approach also makes it easy to compare groups with regard to their latent trait means and the amount of latent trait and state residual variability. It should be noted that the present MP-LST approach can also be used in single-group designs (e.g., when there is no control group), for example, to study whether smokers' affect variability varies across certain situations (e.g., in smoking vs. non-smoking situations).

Previous research has shown that NRT can help improve mean levels of positive affect after quitting relative to placebo (Shiffman et al., 2006). In the present study, we had specific hypotheses about the effects of quitting smoking and the NRT intervention on individuals' affect *variability*. We expected that smokers' affect levels would become more volatile after quitting, leading to increased state variability in the post-quitting as compared to the pre-quitting phase. Furthermore, we expected the NRT intervention to decrease the amount of intra-individual affect variability relative to placebo treatment after quitting. MG-MP-LST models are perfectly suited for testing hypotheses of this kind, because they allow researchers to distinguish between trait, state residual, and measurement error sources of variability, to consider multiple groups simultaneously (here: NRT vs. placebo), and to compare different phases (here: pre vs. post quitting) within and across groups.

Contrary to our hypotheses, we found that the amount of state variability in smokers' self-reported affect states was significantly *decreased* in both the NRT and the placebo group after quitting. Furthermore, we found that the items *calm* and *content* (but not the item *happy*) showed significantly increased trait variances after quitting in both groups. This indicated that smokers' affect states tended to be more consistent and *less* variable (intra-individually) after they quit smoking. Although the groups differed significantly in their affect trait means post quitting (with higher trait means in the NRT group), we did not find a significant difference between the groups with regard to trait and state residual *variances* post quitting. Whereas it was expected that withdrawing from smoking would make mood more volatile, the data showed that mood actually became more stable, perhaps reflecting smokers anhedonic state during initial abstinence. Epping-Jordan et al. (1998) have demonstrated that nicotine withdrawal can raise the reinforcement threshold, making activities less reinforcing and perhaps thus limiting the ability of activities and contexts to raise mood, resulting in less variability.

An alternative hypothesis to explain the reduced variability in self-rated mood after quitting is that participants engaging in the many assessments required by EMA may develop stereotypic response styles after repeated assessments. Such a response bias could have led to seemingly more “consistent” responses in the second half of the data collection. To test this alternative hypothesis, we applied single-group MP-LST models to another EMA dataset in which smokers were not trying to quit, but simply completed assessments over 3 weeks, with no intervention or change in smoking status (for details on this non-quitting data set, see Shiffman et al., 2014). In these data, we did not find a significant reduction in smokers' affect state variability when examining a similar time frame as in the main study, although the items *happy* and *calm* showed significant increases in trait variance (26 and 27%, respectively) across time (the item *content* was not assessed in that study), leading to higher consistency and lower occasion-specificity values in the second half of the study.

In the quitting data example reported in the present paper, we observed a significant and large (29%) reduction in state variability from the pre- to the post-quitting phase in both the NRT and placebo groups. Moreover, in the quitting study, the increases in trait variance for *calm* (58%) and *content* (45%), but not happy, were substantially larger than in the data set without quitting. Taken together, these results suggest that quitting smoking itself may indeed have an effect to reduce state variability in positive mood above and beyond potential response style effects. This could be due to repeated smoking, which leads to large fluctuations in nicotine levels as nicotine concentrations spike immediately after smoking and then decline with nicotine clearance. However, additional studies are needed to confirm this effect. In addition, alternative explanations for the observed effects that we cannot fully rule out may include maturation and testing effects.

### Relations to other methods

The MG-MP-LST models presented and applied in the present paper are closely related (but not identical) to models discussed by Eid and Hoffmann (1998) as well as Steyer et al. (2015). Whereas Eid and Hoffmann (1998) as well as Steyer et al. (2015) focused on the use of LST models in the assessment of mean changes and/or causal effects, the present paper focuses on the use of such models for examining changes in intra-individual state variability from pre- to posttest in an experimental setting.

Hedeker et al. (2012; see also Hedeker et al., 2009) recently presented a multilevel modeling approach to studying intra-individual variability in EMA data. One difference between Hedeker et al.'s approach and the LST approach presented here is that the LST approach uses *multiple* indicators and latent variable variance components that are corrected for random measurement error, whereas Hedeker et al.'s approach uses single indicators and assesses variability in the error variables. Hence, Hedeker et al.'s approach does not separate true state variability from random error variability. In contrast, the LST approach uses multiple indicators and therefore enables researchers to explicitly distinguish between trait latent variables (reflecting true person effects), state residual latent variables (reflecting true situation and/or person-situation interaction effects), and measurement error variables (reflecting random measurement error). The LST approach thus allows separating true state variability from random measurement error. An advantage of the LST approach is therefore that it clearly separates *systematic* variability in individuals' responses from unreliable error variability. Moreover, the LST approach allows considering multiple items or scales simultaneously, whether these indicators measure a single unidimensional trait (STMS approach) or multiple distinct facets of one or several traits (MTMS approach as illustrated here). This allows researchers to study and compare multiple related items or other indicators simultaneously and to assess ME across groups and phases. The LST approach also allows researchers to relate both trait and state residual components to external variables (e.g., grouping variables as in the present paper).

Furthermore, the LST approach allows researchers to include systematic autoregressive effects among latent state residual variables if necessary (Cole et al., 2005). Such short-term stability effects must be expected in intensive longitudinal studies that use very small intervals between measurement occasions (such as just a few hours). In the present study, we tested for autoregressive effects, but found those to be non-significant so that we could use simpler models without these effects.

The LST approach also allows researchers to test for ME within and across phases as well as between groups. This is another advantage compared to (M)AN(C)OVA and other more conventional statistical approaches that implicitly make the assumption of ME, but do not allow testing it. In our application, the item *content* showed decreased state residual factor loadings in the placebo group in the post-quitting phase as compared to the pre-quitting phase in the same group. This may indicate that the state residual components (i.e., the occasion-specific deviations of the state from the trait scores) for the item *content* were to a lesser extent shared with the two other items (*happy* and *calm*) post quitting in this group. One might speculate that the experience of contentment relates more strongly to the experience of reinforcement and is thus more affected when nicotine withdrawal is more intense.

In the present case, a sufficient level of partial ME between pre- and post-quitting phase could still be established (Byrne et al., 1989) so that we were able to make meaningful comparisons of state residual factor variances, trait variances, and trait means across groups and phases. Recent techniques for establishing approximate ME (van de Schoot et al., 2013) may also be useful in cases in which full ME cannot be established.

### Limitations

The models discussed here require a relatively large amount of data. Specifically, researchers need to collect data from at least two items or scales on at least two measurement occasions in at least two groups (1) pre and (2) post treatment. This is the minimal design for estimating the described models. We would generally recommend using more indicators and time points within each phase if possible in order to prevent potential model identification and estimation problems and to obtain more dependable parameter estimates. In addition, although it is generally difficult in structural equation modeling to make general sample size recommendations, the required sample size for complex latent variable models such as the MG-MP-LST models is clearly larger than for observed variable methods such as (M)AN(C)OVA. The large amount of required data (especially with regard to the overall sample size) can place a burden on the investigator and may not always be realistic to achieve. An alternative to the described models for smaller sample sizes could be Hedeker et al.'s (2012) multilevel approach, which does not rely on latent variables and may thus be more appropriate for smaller samples than the LST approach.

The approach with indicator-specific trait factors presented here is flexible in that it allows researchers to use and study a set of potentially heterogeneous indicators. Each indicator can be studied separately in terms of both mean changes and changes in state variability. One drawback of this flexibility is that researchers may sometimes find it difficult to come up with specific hypotheses for each indicator. Instead, they may wish to study only a single “general” trait factor. When researchers are interested in studying a single general trait factor, other versions of the basic LST model can be adapted for an MG-MP-LST design. For example, Eid et al. (1999) as well as Geiser and Lockhart (2012) described an LST model with a general trait factor and *I* – 1 specific (residual method) factors to model indicator-specificity (where *I* indicates the total number of indicators to be modeled). Eid et al.'s (1999) model is an alternative to the indicator-specific approach presented here when researchers want to study general trait factors. Their approach can be easily adapted to an MG-MP-LST design.

## Conclusions

MG-MP-LST models are comprehensive longitudinal models that allow experimental and quasi-experimental researchers to test a wide variety of hypotheses with regard to group differences and treatment effects. Many researchers who use (quasi)experimental designs may find MG-MP-LST models to be particularly useful in cases in which interventions, experimental manipulations, or other treatments are hypothesized to affect individuals' intra-individual variability levels—for example, when treatments are expected to have a “stabilizing” effect in terms of reducing intra-individual state variability.

## Author contributions

CG ran statistical analyses and wrote most parts of the manuscript. DG ran statistical analyses, prepared tables and figures, and wrote sections of the manuscript. SS provided the data used for the empirical illustration of the models and edited multiple versions of the manuscript.

## Funding

Research reported in this publication was supported by the National Institute on Drug Abuse of the National Institutes of Health under award number 1R01 DA06084 (Principal Investigator: SS). The content is solely the responsibility of the authors and does not necessarily represent the official views of the National Institutes of Health.

### Conflict of interest statement

The authors declare that the research was conducted in the absence of any commercial or financial relationships that could be construed as a potential conflict of interest.

## Footnotes

^1^With “systematic intra-individual variability” we mean state variability that cannot be explained by random measurement error, but is due to situational effects or person × situation interactions in the sense of LST theory (Steyer et al., 1992).

^2^We also conducted invariance tests for the error variances *Var*(*e*_*it*_). Invariance of the error variances across groups and phases could not be established for the present data. Given that invariance of the error variances is not critical for the latent variable parameter comparisons described in this paper we do not report these analyses in detail here.
