# Supplementary material for: Using Multigroup-Multiphase Latent State-Trait Models to Study Treatment-Induced Changes in Intra-Individual State Variability: An Application to Smokers' Affect
Source: Front Psychol. 2016 Jul 22;7:1043. doi: 10.3389/fpsyg.2016.01043 (PMC4956644; doi:10.3389/fpsyg.2016.01043)
Supplement: Supplementary file 1 [file DataSheet1.docx]

**Online Supplemental Material**

**Mplus Script for Specifying Model 15**

TITLE: Multigroup-multiphase (MG-MP-)LST model for 2 groups, 2 phases,

3 time points per phase, and 3 items per time point.

Model with indicator-specific traits

Strong measurement equivalence (ME), except for item

Content in Placebo group

State residual variances set equal across groups

State residual variances allowed to differ between phases

Equal trait variances across groups

Equal trait variances across phases only for Happy

Equal trait means across groups pre-quitting only

Equal trait means pre-post in Placebo group only

Equal trait covariances across groups within each phase

DATA: file = WideFormatDATA.txt;

VARIABLE:

! Item_it

! i = item

! t = time point

names = ID GROUP TX

pLAP1 pCONTIME1 pCRA1 pH1 pCA1 pI1 pM1 pW1 pCO1 pF1 pS1 ! pre time 1 items

pLAP2 pCONTIME2 pCRA2 pH2 pCA2 pI2 pM2 pW2 pCO2 pF2 pS2 ! pre time 2 items

pLAP3 pCONTIME3 pCRA3 pH3 pCA3 pI3 pM3 pW3 pCO3 pF3 pS3

LAP1 CONTIME1 CRA1 H1 CA1 I1 M1 W1 CO1 F1 S1 ! post time 1 items

LAP2 CONTIME2 CRA2 H2 CA2 I2 M2 W2 CO2 F2 S2 ! post time 2 items

LAP3 CONTIME3 CRA3 H3 CA3 I3 M3 W3 CO3 F3 S3; ! time post 3 items

grouping = TX (0 = Placebo 1 = NRT); ! NRT = nicotine replacement therapy

usevariables =

pH1 pCA1 pCO1 ! happy, calm, content, Phase 1 (Pre quitting), Time 1

pH2 pCA2 pCO2 ! happy, calm, content, Phase 1 (Pre quitting), Time 2

pH3 pCA3 pCO3 ! happy, calm, content, Phase 1 (Pre quitting), Time 3

H1 CA1 CO1 ! happy, calm, content, Phase 2 (Post quitting), Time 1

H2 CA2 CO2 ! happy, calm, content, Phase 2 (Post quitting), Time 2

H3 CA3 CO3; ! happy, calm, content, Phase 2 (Post quitting), Time 3

MODEL:

! PRE-QUITTING PHASE (PHASE 0)

! Item-specific trait factors PRE (T_i0)

! Trait for Item 1 (happy)

T10 by pH1@1

pH2@1

pH3@1;

! Trait for Item 2 (calm)

T20 by pCA1@1

pCA2@1

pCA3@1;

! Trait for Item 3 (content)

T30 by pCO1@1

pCO2@1

pCO3@1;

! State residual factors PRE (SR_t0)

! set factor loadings (gamma) equal across time

SR10 by pH1@1

pCA1 (gamma2)

pCO1 (gamma3);

SR20 by pH2@1

pCA2 (gamma2)

pCO2 (gamma3);

SR30 by pH3@1

pCA3 (gamma2)

pCO3 (gamma3);

! Set all item intercepts to zero

[pH1-pCO3@0];

! Non-admissible correlations.

T10-T30 with SR10-SR30@0;

SR10-SR30 with SR10-SR30@0;

! POST-QUITTING PHASE (PHASE 1)

! Item-specific trait factors POST (T_i1)

! Trait for Item 1 (happy)

T11 by H1@1

H2@1

H3@1;

! Trait for Item 2 (calm)

T21 by CA1@1

CA2@1

CA3@1;

! Trait for Item 3 (content)

T31 by CO1@1

CO2@1

CO3@1;

! State residual factors POST (SR_t1)

! set factor loadings (gamma) equal across time

SR11 by H1@1

CA1 (gamma2)

CO1 (gamma3P);

SR21 by H2@1

CA2 (gamma2)

CO2 (gamma3P);

SR31 by H3@1

CA3 (gamma2)

CO3 (gamma3P);

! Set all item intercepts to zero

[H1-CO3@0];

! Non-admissible correlations.

T11-T31 T10-T30 with SR10-SR30@0 SR11-SR31@0;

SR11-SR31 SR10-SR30 with SR11-SR31@0;

! GROUP-SPECIFIC STATEMENTS

! Placebo group

Model Placebo:

! Estimate latent trait means PRE (set equal across groups)

[T10] (T10mean);

[T20] (T20mean);

[T30] (T30mean);

! Estimate latent trait means POST (set equal to pre-quitting means)

[T11] (T10mean);

[T21] (T20mean);

[T31] (T30mean);

! Set state residual variances equal within the pre-quitting phase

SR10 SR20 SR30 (SRvarPRE);

! Set state residual variances equal within the post-quitting phase

SR11 SR21 SR31 (SRvarPOS);

! Trait variances (set equal across groups)

T10 (T1var); ! Happy variance also set equal across phases

T20 (T20var);

T30 (T30var);

T11 (T1var);

T21 (T21var);

T31 (T31var);

! Trait covariances (set equal across groups)

T10 with T20 (cov120);

T10 with T30 (cov130);

T20 with T30 (cov230);

T11 with T21 (cov121);

T11 with T31 (cov131);

T21 with T31 (cov231);

! Nicotine replacement therapy (NRT) group

Model NRT:

! Set state residual factor means to zero

[SR10-SR31@0];

! Set state residual factor loadings (gamma) equal across time and phases

SR10 by pH1@1

pCA1 (gamma2)

pCO1 (gamma3);

SR20 by pH2@1

pCA2 (gamma2)

pCO2 (gamma3);

SR30 by pH3@1

pCA3 (gamma2)

pCO3 (gamma3);

SR11 by H1@1

CA1 (gamma2)

CO1 (gamma3);

SR21 by H2@1

CA2 (gamma2)

CO2 (gamma3);

SR31 by H3@1

CA3 (gamma2)

CO3 (gamma3);

! Set SR variances equal within the pre-quitting phase

! and across groups

SR10 SR20 SR30 (SRvarPRE);

! Set SR variances equal within the post-quitting phase

! and across groups

SR11 SR21 SR31 (SRvarPOS);

! Estimate latent trait means PRE (set equal across groups)

[T10] (T10mean);

[T20] (T20mean);

[T30] (T30mean);

! Estimate latent trait means POST (not set equal across groups)

[T11] (T11mA);

[T21] (T21mA);

[T31] (T31mA);

! Trait variances (set equal across groups)

T10 (T1var); ! Happy variance also set equal across phases

T20 (T20var);

T30 (T30var);

T11 (T1var);

T21 (T21var);

T31 (T31var);

! Trait covariances (set equal across groups)

T10 with T20 (cov120);

T10 with T30 (cov130);

T20 with T30 (cov230);

T11 with T21 (cov121);

T11 with T31 (cov131);

T21 with T31 (cov231);

OUTPUT: sampstat stdyx;
